# Experiences of Filipino Americans with Type 2 Diabetes during COVID-19: A Qualitative Study

**DOI:** 10.1177/01939459231162917

**Published:** 2023-03-21

**Authors:** Dante Anthony Tolentino, Rey Paolo Ernesto Roca, Joey Yang, Josephine Itchon, Mary E. Byrnes

**Affiliations:** 1 School of Nursing, University of California Los Angeles, CA, USA; 2 Undergraduate Research Opportunity Program, University of Michigan, Ann Arbor, MI, USA; 3 Department of Surgery, University of Michigan, Ann Arbor, MI, USA

**Keywords:** Filipino Americans, type 2 diabetes mellitus, COVID-19, qualitative research, health equity

## Abstract

Little is known about the experiences of Filipino Americans with type 2 diabetes regarding their self-management during the early phase of the COVID-19 pandemic. We conducted a qualitative research study using semistructured interviews. In total, 19 interviews were recorded, transcribed, and analyzed by 4 independent coders. We situated our understanding of these results using three concepts from an indigenous Filipino knowledge system called *Sikolohiyang Pilipino: Kapwa* (shared identity), *Bahala Na* (determination), and *Pakikibaka* (spaces of resistance). The following three main themes emerged: (1) stressors of the pandemic, (2) coping behaviors (with two subthemes: emotional and lifestyle-focused responses), and (3) diabetes self-management outcomes. Participants experienced stresses, anxiety, and loneliness during the pandemic magnified by the complexities of self-management. Although many admitted the pandemic brought challenges, including burnout, they coped by using existing resources—support from family, friends, the use of technology, and various emotional coping mechanisms. Many said that they made few diabetes self-management changes.

Type 2 diabetes (T2D) disproportionately affects historically and contemporary marginalized groups,^1^ including Filipino Americans. T2D among Filipino Americans is prevalent (10.2% to 19.4%)^2,3^ with evidence suggesting that Filipino Americans have suboptimal self-management behaviors on healthy eating, medication taking, and blood glucose testing.^4^ With many struggling to cope with self-management under normal circumstances, the novel SARS Coronavirus 2019 (COVID-19) pandemic added to an already complex management of T2D. Filipino Americans are disproportionately dying from COVID-19, accounting for 30% of deaths in California.^5^ Diabetes is also associated with a higher risk of a more severe course of COVID-19 and long-term complications.^6^ Despite the high prevalence of T2D and increased mortality rates among Filipino Americans during COVID-19, they continue to be understudied in the United States.^7^

## Epistemic and Ontologic Understanding of Filipino American Health

While experiences of individuals with T2D during the pandemic have been studied in the last two years,^8-12^ many focused on populations outside of the United States and overlooked the perspectives of Filipino Americans. Much of the extant literature on marginalized groups has also used Western epistemic and ontologic understandings of health and illness, wherein health is built on a biomedical model that addresses health deficits and disease symptoms and often gives little regard to sociocultural influences. Due to the lack of T2D studies focused on Filipino Americans, health outcomes and experiences are often invisible or misunderstood. For studies that include Filipino Americans, many undermine the influence of generational trauma, racism, and inaccurate risk factors.^7^ Given the multifaceted impact of sociocultural and historical factors, and with the increase in decolonizing health research that requires addressing effects of colonization, structural oppression, and disenfranchisement,^7^ we examine Filipino American experiences with T2D during the early phase of the pandemic with a strength-based lens using an indigenous knowledge system called *Sikolohiyang Pilipino*.^13-15^*Sikolohiyang Pilipino* (Filipino psychology) refers to the psychology drawn from the Filipino people’s own experiences, thoughts, and perspectives.

A dominant narrative among Filipino American health studies is the concept of resiliency.^16-18^ Although essential, the resiliency narrative is often grounded in Western health conceptualizations and detracts from understanding how inherent Filipino values impact diabetes self-management. We grounded our discussion of the results using *Sikolohiyang Pilipino* to reframe this narrative.^13^ We used this critical approach using the concepts of *kapwa*, *pakikibaka*, and *bahala na* and how they relate to the experiences of Filipino Americans with T2D during the early phase of the pandemic.

### Overview of Sikolohiyang Pilipino

Due to the American colonization of the Philippines, Filipinos are typically characterized using Western philosophical conceptions. Influenced by colonialist worldviews, these Western concepts reinforce the idea of Filipino inferiority to colonizers, many of which are not culturally concordant.^19^ For instance, many studies of chronic diseases, including T2D, focus on biomedical factors of diagnosis, management, and outcomes, neglecting sociocultural dimensions.^20^ These Western views fail to recognize how Filipinos define themselves (i.e., the true spirit of Filipinos) and contribute to the formation of negative legacies of historical trauma, colonial mentality, and poor physical and mental health.^21^*Sikolohiyang Pilipino* frames and addresses these challenges. It examines historical and sociocultural facts about Filipinos, an understanding of the indigenous language, the uncovering of Filipino practices and values, and the interpretation of such characteristics from the standpoint of the indigenous Filipino conscience, knowledge, habits, and behaviors.^13,15^

We used three main concepts from *Sikolohiyang Pilipino* in this study. First is the concept of *kapwa*, or shared identity. In *kapwa*, individuals recognize that they share a fundamental nature with others.^13^ The unity of one’s self with others implies inclusiveness and a sense of shared space and humanity by establishing a connection. The second concept is *pakikibaka*, or spaces of resistance. *Pakikibaka* is the ability of Filipinos to undertake internal and external challenges and the effort to adapt and work to bring about necessary changes in systems.^13^ Finally, we used the concept of *bahala na*, or determination. Although often referred to as a fatalistic attitude, in *Sikolohiyang Pilipino bahala na* is an attitude of “determination and risk-taking.”^13^*Bahala na* is not passively accepting what is to come; rather, it is a conviction that they can overcome challenges or barriers.

## Purpose

This study aimed to understand Filipino Americans’ behaviors, values, perceptions, and challenges related to T2D self-management during the early phase of the COVID-19 pandemic, grounding the discussion of the results in *Sikolohiyang Pilipino*, an indigenous Filipino psychological framework.

## Methods

### Study Design

We used interpretive description, a noncategorical qualitative methodology to understand the experiences of Filipino Americans with T2D, including their values, perceptions, and challenges with T2D self-management during the first wave of COVID-19.^22,23^ Interpretive description allows us to understand how Filipino Americans experience their health and illness by considering the constructed and contextual circumstances allowing for shared realities. The study was approved by the University of Michigan Institutional Review Board (HUMID #00194036). Informed consent was obtained from all study participants.

### Researcher Characteristics

The first author, D.A.T., is a PhD-prepared Filipino American nurse who emigrated from the Philippines at the age of 18. The first author has more than a decade working as a nurse informaticist, with a specific research interest in T2D among Filipino Americans. He is trained in qualitative research. The second author, R.P.E.R., is also a Filipino American nurse with research experience in illness perception and T2D among Filipino Americans. He is a seasoned nurse educator, has worked alongside the Filipino American community in the last 15 years, and is currently finishing his PhD studies. Authors J.Y. and J.I. are undergraduate students and are learners of qualitative research who added reflexivity into the analysis. The senior author, M.B., is a scholar of sociology with content expertise in intersectionality and inequalities and methodological expertise in qualitative methods.

### Study Population

To reach a broad range of Filipino Americans with T2D, we leveraged the strengths of social media (e.g., Facebook, Twitter, Instagram) to purposively sample potential participants from March to August 2021. Participants self-identified as Filipino American, >18 years old, understood English, and were diagnosed with T2D > 1 year (self-identified in the eligibility survey). In total, 37 participants completed the eligibility survey, and 19 participants completed the study and were compensated with a $25 gift card.

### Data Collection

We collected demographic information using an online survey (Qualtrics, Provo, UT) with potential participants consenting to a virtual interview. The first and last authors (D.A.T. and M.E.B.) conducted independent one-on-one semistructured virtual interviews over a secure video conference platform from March 2021 to August 2021. Informed consent was obtained verbally prior to the start of the interview. The interview guide was intended to elicit in-depth responses on their T2D self-management experiences during the pandemic. Interviews ranged from 35 to 90 minutes and were digitally recorded, professionally transcribed verbatim, and redacted for identifying information. D.A.T. reviewed the transcripts for accuracy and anonymity. Unique identifiers were applied to each participant for referencing purposes and to protect confidentiality.

### Data Analysis

We approached our initial coding through an iterative, inductive process. The initial codebook was created by three authors (D.A.T., J.I., and J.Y.) reviewing transcripts to identify an initial set of codes. The codes were refined through discussions, adding a fourth author (R.P.E.R.). Four members independently coded the transcripts deductively utilizing the initial codes, and then independently performed emotions and values coding inductively.^24^ The team then organized the coded segments into categories, subthemes, and themes. We reached information power with a sample size of 19, as determined by our narrow aim and quality of dialogue that produced information-rich cases.^25^

#### Sikolohiyang Pilipino

To shift the deficit-based paradigm of discussing Filipino American health, we used Enriquez’ *Sikolohiyang Pilipino* (Filipino psychology) as a form of a framework to interpret the findings^26^ using the indigenous concepts of Filipino behavior patterns and value structure.^13^ We used three concepts described in *Sikolohiyang Pilipino*: the core value of (1) *kapwa* and confrontative surface values of (2) *bahala na* and (3) *pakikibaka*, to situate the results of our findings.^13^

## Results

### Participant Characteristics

Table 1 displays the sociodemographic characteristics of the participants. About 90% of the 19 participants were born in the Philippines, 69% identified as women, and 5% were nonbinary. The mean age was 57.3 years (*SD* = 13.8). More than 68% had a bachelor’s degree. Over 70% lived in California, and 53% had an annual income of > $60,000. All participants had health insurance, a primary care provider, and no hospitalizations in the previous year. Many self-reported comorbidities include obesity, sleep apnea, hypertension, hypercholesteremia, gout, and arthritis.

**Table 1.** Participant Characteristics (*N* = 19).

Characteristic	*n* (%)
Age	Mean = 57.32 (*SD* = 14.22)
<40 years old	1 (5.3%)
41-50 years old	3 (15.8%)
51-60 years old	6 (31.6%)
61-70 years old	3 (15.8%)
>71 years old	5 (26.3%)
Birthplace	
Philippines	17 (89.5%)
United States	2 (10.5%)
Education	
Some college	2 (10.5%)
Associate degree	3 (15.8%)
Bachelor’s degree	6 (31.6%)
Master’s degree	6 (31.6%)
Doctoral degree	1 (5.3%)
Gender	
Nonbinary	1 (5.3%)
Woman	13 (68.4%)
Man	5 (26.3%)
Income	
<$30K	2 (10.5%)
$31K-$60K	3 (15.8%)
$61K-$100K	5 (26.3%)
$76K ≥ $100K	7 (36.8%)
Location	
California Nevada	14 (74.0%) 1 (5.2%)
Georgia	1 (5.2%)
Indiana	1 (5.2%)
New York	1 (5.2%)
Ohio	1 (5.2%)
Job	
Nurse	6 (31.6%)
Administrative (e.g., banking)	5 (26.3%)
Sales and marketing	1 (5.3%)
Retired	6 (31.6%)
Academia	1 (5.3%)
Has primary care provider	19 (100%)
Has health insurance	19 (100%)
Last HbA1C	
<7%	10 (52.7%)
7-8%	6 (31.6%)
>8.1%	3 (15.8%)
Years with type 2 diabetes	
1-10 years	12 (63.2%)
11-20 years	4 (21.0%)
>20 years	1 (5.3%)

### Themes

**Figure 1.**
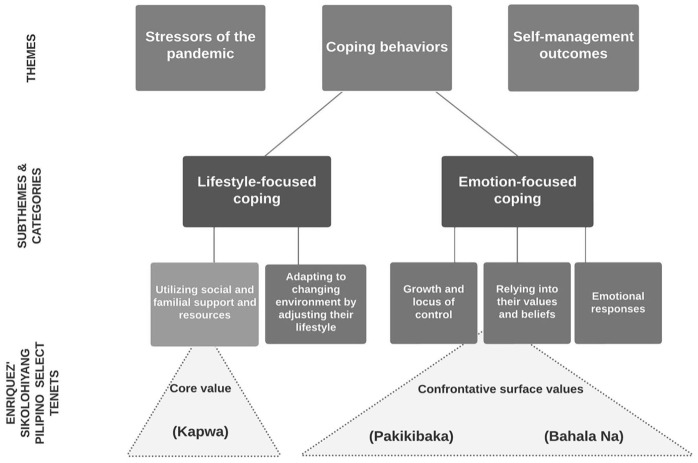
Key Themes and Subthemes and How Select Tenets of Sikolohiyang Pilipino Overlays with the Coping Behaviors Subthemes/Categories.

Figure 1 displays the themes identified from the analysis. Key themes are as follows: (1) stressors of the pandemic; (2) coping behaviors related to the pandemic; and (3) self-management outcomes. The three concepts from Enriquez’ *Sikolohiyang Pilipino* overlap the five categories that emerged from the coping behaviors theme.

#### Stressors of the pandemic

Participants described everyday pandemic stressors, regardless of their occupation during the early phase of the pandemic. While many were essential workers (e.g., nurses and frontline workers), a common theme emerged in that they talked about work-related issues that brought stress or anxiety, including changes in work structure (e.g., irregular hours, remote work), job duties, and insufficient work training/resources. A nurse described her stress related to hospital work and the inability to go home to her family: “I wasn’t managing [referring to T2D] because the stress was all the way up there. Plus, you can’t go home” (P19, 60 years old). Some participants mentioned how changes in their job structure contributed to their stress throughout the early phase of the pandemic. One participant said, “I’ve worked through the pandemic. I. . .never had time off because I work at a bank, so we have to support finance, which was hard. . .now I’ve been taking sick days, like mental days” (P11, 51 years old).

Many described different emotions, (i.e., feelings of depression, loneliness, isolation, or anxiety). Pandemic restrictions, like lockdowns, contributed to depression. One participant shared: “Something COVID brought to me, which I must confess. It may have added to my depression. If I am a depressed person—totally you’re like in prison. I could hardly move and see the sun, and you’re limited” (P8, 82 years old). Others felt lonely because they worked from home while their significant others had to go to the office. Due to loneliness, a participant turned to food as comfort: “I still eat the same amount of rice. I don’t know why. Maybe because of COVID. There was a time that—I don’t wanna say—I get sad. . .food was my comfort” (P6, 44 years old).

The inability to go out and exercise also contributed to feelings of sadness. One simply desired to go outside, saying "I was actually in a gym, health club before COVID started, and I just felt myself just wearing down, just gloomy from lack of exercise” (P18, 54 years old).

Other pandemic stressors magnified the complexities of T2D self-management. These stressors included school, family, and comorbidities. When asked about how COVID-19 impacted their self-management, one participant expressed, “divorce, finals, midterms. Those are the things that stress me out, but not COVID-19. I will not get stressed out with that” (P4, 55 years old).

#### Coping behaviors during the pandemic

The pandemic evoked feelings of isolation, shock, and confusion; consequently, participants developed patterned responses to cope with the scale and unpredictability of the pandemic. The following two subthemes emerged: (1) lifestyle-focused coping and (2) emotion-focused coping.

We used three indigenous concepts from *Sikolohiyang Pilipino* that overlapped with the theme of coping behaviors to explain these findings. First is the concept of *kapwa*, a core value of Filipino indigenous psychology that stresses the interconnectedness with one another.^13^ Second is the indigenous concept of *pakikibaka*. This concept refers to an internal conflict with a concerted effort to adapt which can be accomplished by thriving in a challenging environment or for some, failing to adapt. Finally, the concept of *bahala na* is a Filipino sociocultural value translated to “whatever will be, will be.” It is, however, more than a fatalistic attitude; it is about determination in the face of uncertainty.^13^

##### Lifestyle-focused coping

Lifestyle-focused coping centered around participants’ adaptation to their changing environment by using social/family support and resources and adjusting their lifestyle related to diet and exercise.

##### Utilizing social and familial support and resources

In this study, *kapwa* was represented by their family’s role in their T2D self-management to cope during the pandemic. Many relied on their family as their social support. One participant said of her family, “They were very concerned. . .so they encouraged us to have our groceries delivered, so we don’t go out” (P17, 69 years old). Some family members also encouraged them to exercise. A participant recalled that their husband said, “Come on, you need to walk. We haven’t walked for a day” (P16, 70 years old).

Technology also played a role in helping participants find new ways to connect with their social support and needed resources (e.g., health care needs) by turning to video communications technology (e.g., Zoom^®^). Some noted using telehealth to meet with their care provider, while others used virtual platforms for their physical activities. One participant spoke about using online Zumba, “I just look for Zumba classes. . .. It worked out okay. I could do it whenever. . .it fitted my schedule very well” (P17, 69 years old). Another talked about her husband buying her a smartwatch, saying “My husband bought this for me [pointing to smart watch], so I can monitor my lifestyle. We did go to the gym but ever since the pandemic, we couldn’t” (P16, 70 years old).

##### Adapting to a changing environment by adjusting their lifestyle

The concept of *pakikibaka* closely aligns with this category. This relates to the pandemic-induced changes in participants’ eating patterns and physical activities—some experienced positive changes in their eating habits, while some may have had negative adaptations. Before the pandemic, some shared that they would always eat out. However, the lockdowns during the pandemic helped them manage their unhealthy eating habits. One participant said, “It helps me manage eating out, like a bad eating habit, there’s a lot of fast food [prior to COVID] . . .I barely ate out because I couldn’t eat out” (P3, 68 years old). Another remarked that the pandemic forced them to change their poor eating habits: “Well, this pandemic has some good effect on this diabetes thing. If there is no pandemic, we’ll be traveling and eat, eat, eat. . .we cannot do that because of the pandemic. So it helps” (P9, 71 years old).

Others, however, failed to make some positive changes in their eating habits. This was because they would eat what was available, even if it was unhealthy. This form of *pakikibaka* was noted by a participant:

I’m at home with my family. . .my dad, who cooks whatever food he wants, then I will eat with them. So we had a lockdown, like [in March]. So around December, I was already gaining weight, although my blood sugar was still okay (P15, 54 years old).

Others spoke about missing the opportunity to do in-person exercises because of lockdowns. However, this did not stop them from making healthy choices. Instead, they resisted being sedentary by acknowledging deficiencies and making adjustments. One said, “When I used to go to the office during my lunch or break, I would walk around. But due to the pandemic, just staying home, I only exercise during my lunchtime. I don’t think it’s enough, the 30 minutes cardio” (P6, 44 years old).

Virtual events helped many adapt. One Silver Sneakers participant detailed how she continued her fitness regimen during the pandemic: “I Googled Zumba online and I was just able to do it. Nobody cares about how you look. . .so I just look for Zumba classes. . .. Not the real fancy ones but just ones that are more like less jumping” (P17, 60 years old).

##### Emotion-focused coping

Most participants exhibited emotion-focused coping during the pandemic. These were strategies that regulated their emotional reactions to the pandemic. There was a spectrum of growth and control, values and beliefs reliance, and emotional responses. The concepts of *pakikibaka* and *bahala na* were exhibited in many of the emotion-focused coping strategies manifested by the participants, particularly in growth and in relying on their values and beliefs.

##### Growth and locus of control

This reflects *pakikibaka*, the capacity to thrive despite the pandemic due to their internal locus of control, believing that their self-management behaviors are determined by their own decisions and efforts. A participant talked about her experience wearing a mask—a behavior that she could control during the pandemic: “I’m very careful not just because of the diabetes, but I also have asthma. I wear a mask all the time. . .. Even if they say stop using it, I’m still using it” (P19, 60 years old).

Despite the pandemic’s stressors, numerous participants noted self-growth, including learning new skills like cooking and gardening. One said, “I learned to cook something. If I’m craving something. . .I have to research the internet, and then there you go, there’s the recipe” (P7, 51 years old).

##### Relying on their values and beliefs

Participants turned to their values and beliefs as coping strategies during the pandemic. Many described their belief in determination and risk-taking (*bahala na*). One participant explained that while he had friends who smoked, they have not been diagnosed with lung cancer. He explained that it is about living life and that medicine will have an answer. He described it as,

If you are going to look at each statistic, the lifespan of man has increased almost threefold from just, uh. Now, people can live longer. . .and so, the diabetes I think, uh, modern medicine will be able to cure diabetes. (P8, 82 years old)

Similarly, another talked about how they put their lives in God’s hands after contracting COVID-19: “I am not really afraid to transition. You know? Whatever happens to me, it is what it is. It’s God’s will” (P1, 42 years old). The participant later explained that he thinks about the present rather than the future. “I’m worried about is what’s now, what’s happening now rather than what’s gonna happen in the near future” (P1, 42 years old).

##### Emotional responses

Many exhibited emotional reactions related to their pandemic experiences, including being scared, anxious, frustrated, and cautious. One said, “The initial scare of COVID is because I am a higher risk so going to work. . .I was very anxious” (P5, 47 years old). Many were cautious in meeting people outside of their household, and some took extra sanitizing measures. One said, “When my daughter had her vaccine—every time she come to my house, I said, You have to take a bath first. I wear a mask when some come in” (P3, 68 years old).

Others expressed their frustration because of the imposed restrictions. Another lamented not being able to return to the Philippines, saying “the only thing might be my frustration. We cannot travel. We were supposed to go to the Philippines about earlier part of this year” (P13, 86 years old).

#### Self-management outcomes

Despite the pandemic’s stresses, more than half acknowledged they made minor adjustments to their T2D self-management activities. Many said, "no changes” (P2, 36 years old), "not much changed” (P13, 86 years old), or "same thing” (P12, 65 years old). One admitted that "nothing has really changed as far as the COVID. I’m pretty much doing the same thing I’ve been doing pre-COVID” (P18, 54 years old).

Although many admitted no changes in behaviors, some expressed T2D self-management burnout (i.e., struggling with blood sugar and weight management), admitting exhaustion. One said, “With the remote work, I gained weight like everybody else” (P15, 54 years old). Another talked about how feelings of loneliness have led to burnout. She said, “I would feel sad like if my sugar’s elevated like I don’t care. And then I stop checking my glucose every day. . .it’s just like why am I doing this?” (P6, 44 years old).

Some missed medical appointments due to the pandemic. One participant described the challenges of making appointments during the pandemic, saying “I choose not to go. Like last year, I missed the physical because of the whole COVID thing” (P11, 51 years old). Some found the shift in their routine demanding. One participant who worked half a day to care for their children said self-management was impaired:

It changed, actually. Because last year, with the pandemic, I had to take a half-day off at work, for the kids" and because of the challenges with the pandemic she was not able to “do the right diet, it’s hard with three kids goin’ to work and then this pandemic” (P2, 36 years old).

Another person admitted that the pandemic was an unexpected change that shifted their way of thinking and behaving related to T2D self-management. They said,

It wasn’t easy, because you know, at my age, you have to do a lot of behavior modification. We have to figure out what can we do differently? We thought this was gonna go away in a couple months (P17, 69 years old).

## Discussion

Filipino Americans in our study described shared experiences of living with T2D during the early phase of the COVID-19 pandemic, including pandemic stressors, emotional responses and coping, and the consequence of COVID-19 on T2D self-management outcomes. Similar to prior studies,^9,11,12,27,28^ negative emotions compounded the stress many people felt due to the pandemic’s uncertainty, which led to difficulty in T2D self-management. However, framing these results exclusively using a deficit-based lens (e.g., negative emotions and outcomes brought by the pandemic) fails to recognize the complex sociocultural realities of Filipino Americans and diminishes their indigenous values.^13^ Therefore, we situated this discussion using the core value of *kapwa* and the confrontative surface values of *pakikibaka* and *bahala na* to explain our findings.

As a core value, *kapwa* (translation: “together with the person” or “shared identity”) is about strengthening and preserving relationships.^14^ It maintains a connection and sense of community, which lies deep within Filipinos’ psyche.^13^*Kapwa* is a predominant Filipino value in which family or friends are considered *hindi ibang tao* (one of us). Unlike other studies where lack of social/family support was one of the barriers during the pandemic,^28^ this community connection was evident in that family and social support played a critical role in their daily management of T2D. Many family members functioned as their care providers and health coaches, providing advice and urging them to exercise and eat better. Due to social distancing measures, many were also forced to turn to collaboration technology for continuity as a lifeline, which enabled them to still share a space with their loved ones and connect with needed resources (such as seeing a health care provider). The value of *kapwa* is reflected as a way of support (*kaakbay*) by one’s kin (*kaanak*) and by others (*kasama*).

As a confrontative surface value, *pakikibaka*, or “resistance,” was evident as a coping mechanism for many participants when dealing with T2D self-management changes. Many resisted in the form of adapting and thriving in a challenging environment. Recent research shows significant declines in physical activity among individuals with T2D during the pandemic.^29^ Yet, in this study, many adapted to the pandemic challenges by being ingenious, such as using previously neglected resources. They also resisted the pandemic challenges by exhibiting control and growth. Many acquired new habits and skills as positive deviance to cope with the pandemic. Another form of adaptation was using technology. People were able to maintain some form of normality in their social lives and received up-to-date information about COVID-19 without placing themselves or their families in danger using digital technology. Despite the severe situation, new technologies presented significant hope for the future by reducing traditional barriers to maintaining social engagement, support exchange, and knowledge collecting.^30^

We found coping behaviors aligned to *bahala na. Bahala na’s* pervasive interpretation of fatalistic resignation originated from Bostrom’s work of American fatalism.^14^ When taken in isolation, automatic resignation may seem apparent in this study; however, participants’ *bahala na* attitude was about determination and risk-taking. Filipinos were assuring themselves that they were prepared to handle the difficult situation before them and would do all possible to achieve their goals by saying *bahala na*. It is not about pessimism but rather a positive affirmation^31^—showing an internal control that they are ready to deal with the challenges of the pandemic—taking the risk even with uncertainty and possible failure.

Filipino Americans are historically understudied, and to the best of our knowledge, this study is the first to examine the experiences of Filipino Americans with T2D during the pandemic. Many of the participants described the challenges and struggles they experienced in T2D self-management during the pandemic, including feelings of stress and anxiety which are echoed in other COVID-19 studies. Filipino Americans adapted to the changing pandemic environment by seeking different ways of exercising (e.g., using technology) and eating (e.g., cooking at home). Many valued the support from their family as a source of *kapwa* in preserving their physical and mental health. Many, however, perceived no changes in most of their self-management behaviors, despite the burden of the pandemic. We also situated our discussion using *Sikolohiyang Pilipino* to ground our understanding of these results as a strength-based rather than a deficit-based model.

There were several limitations to this study. The study was primarily conducted in English, limiting some participants’ expressions of opinions and values. Due to the pandemic, all interviews were conducted online, which may have hampered our ability to assess nonverbal cues that would have provided deeper context for the participants’ responses. The study was also conducted during the early phase of the pandemic; consequently, perceptions and experiences of individuals may have been different from subsequent waves of the pandemic. Although important constructs within a Filipino American context such as acculturative stress and intergenerational family relationships did not emerge as themes in this study, more research is required to uncover the dynamics of these concepts to T2D self-management.

The pandemic subjected different types of stresses among Filipino American individuals with T2D, which has amplified the complexities of T2D self-management. Anxiety, loneliness, and even depression, compounded by the pandemic’s uncertainty, have led to self-management issues. However, many made adaptations in response to the evolving nature of the pandemic. They unraveled how *kapwa*, *bahala na*, and *pakikibaka* can help frame deficit-like experiences as strengths. Clinical implications of this research involve the understanding of how Filipino Americans with T2D manage their self-management behaviors during crisis situations like a pandemic. Examining these values and perceptions could aid in the development of support strategies and standards that are culturally specific. Many of the participants also admitted the feeling of stress and mental health strain associated with the pandemic and living with T2D. With the pervasiveness of stigma surrounding mental health in Filipino American communities, many individuals often avoid seeking help. This study highlights that Filipino Americans can be open to talking about mental health, and clinicians should offer targeted mental health services or interventions to Filipino Americans with T2D. We hope that future research will include more historically marginalized populations in their studies, focus on situating research using culturally relevant theories, and develop interventions that consider lived experiences of their population of interest.
